# Gauging innovation and health impact from biomedical research: survey results and interviews with recipients of EU-funding in the fields of Alzheimer’s disease, breast cancer and prostate cancer

**DOI:** 10.1186/s12961-023-00981-z

**Published:** 2023-06-29

**Authors:** Francesca Pistollato, Ivana Campia, Evangelos P. Daskalopoulos, Camilla Bernasconi, Christian Desaintes, Sergio Di Virgilio, Christina Kyriakopoulou, Maurice Whelan, Pierre Deceuninck

**Affiliations:** 1 European Commission, Joint Research Centre (JRC), Directorate F-Health, Consumers and Reference Materials, Via E. Fermi 2749, 21027 Ispra, VA Italy; 2 European Commission, DG Research & Innovation (DG RTD), Brussels, Belgium

**Keywords:** Alzheimer’s disease, Breast cancer, Prostate cancer, Biomedical research, Impact, EU funding, Indicators, Non-animal models

## Abstract

Biomedical research on Alzheimer’s disease (AD), breast cancer (BC) and prostate cancer (PC) has globally improved our understanding of the etiopathological mechanisms underlying the onset of these diseases, often with the goal to identify associated genetic and environmental risk factors and develop new medicines. However, the prevalence of these diseases and failure rate in drug development remain high. Being able to retrospectively monitor the major scientific breakthroughs and impact of such investment endeavors is important to re-address funding strategies if and when needed. The EU has supported research into those diseases via its successive framework programmes for research, technological development and innovation. The European Commission (EC) has already undertaken several activities to monitor research impact. As an additional contribution, the EC Joint Research Centre (JRC) launched in 2020 a survey addressed to former and current participants of EU-funded research projects in the fields of AD, BC and PC, with the aim to understand how EU-funded research has contributed to scientific innovation and societal impact, and how the selection of the experimental models may have underpinned the advances made. Further feedback was also gathered through in-depth interviews with some selected survey participants representative of the diverse pre-clinical models used in the EU-funded projects. A comprehensive analysis of survey replies, complemented with the information derived from the interviews, has recently been published in a Synopsis report. Here we discuss the main findings of this analysis and propose a set of priority actions that could be considered to help improving the translation of scientific innovation of biomedical research into societal impact.

**Supplementary Information:**

The online version contains supplementary material available at 10.1186/s12961-023-00981-z.

## Background

Alzheimer’s disease (AD), breast cancer (BC) and prostate cancer (PC) have become increasingly prevalent in Europe and worldwide. Nearly 10 million new cases of dementia are recorded every year, with AD contributing to 60–70% of all dementia cases [1], whilst in 2020, BC and PC ranked as the 1st and 4th most common cancer types, respectively [2].

Biomedical research has improved our understanding of the molecular and cellular mechanisms underlying the onset and progression of these complex non-communicable diseases, with the goal to identify associated genetic and environmental risk factors and develop effective and safe treatments to cure or alleviate the burden of these pathologies [3]. Despite research successes, the failure rate in drug development for these diseases remains very high, with more than 95% of tested medicines not receiving regulatory approval [4, 5]. Flaws in the design of animal experimentation, the inappropriate selection of drug targets, overlooking efficacy, pharmacodynamic and pharmacokinetic properties of novel compounds, as well as the inaccurate selection of participants in clinical trials, are considered some of the plausible reasons underlying clinical failure in drug development [3, 6].

In recent years, a wide range of new approach methodologies (NAMs) have been developed, such as cultures using induced pluripotent stem cells (iPSCs) derived from patients [7–9], three-dimensional (3D) tumor spheroids [10], microfluidic organ-on-chip technologies [11, 12], integrated computer modelling, systems biology and imaging techniques [13, 14]. These models and tools, combined with data derived from clinical studies, are used to deepen our knowledge of disease molecular mechanisms, identify new predictive biomarkers, and design novel therapeutic or preventive strategies.

The rise of NAMs in life science, and the increasing need for multidisciplinary approaches, have fostered many research initiatives and the launch of several research projects funded by the European Commission (EC), particularly under Framework Programme 7 (FP7: 2007–2013), and Horizon 2020 (H2020: 2014–2020), to further develop such innovative approaches [15, 16], with substantial funding allocated on AD, BC and PC [3].

The European Union (EU) Framework Programmes (FPs) investments have contributed to key scientific advancements and discoveries and have enhanced our understanding of diseases etiology. Nowadays, there is the need to identify and capture both the direct and indirect effects that such research investments are having over time. However, assessing both the economic and societal impacts of research initiatives and understanding how funded research has been able to address societal needs represent challenging tasks [17]. Moreover, the lack of indicators and suitable methodologies to measure these outputs further complicates these monitoring efforts [18].

Whilst alternative metrics (‘altmetrics’) have been developed to measure societal impact of funded research and assess research outreach on social media and policy documents [19], the dissemination of research outcomes has been questioned as it may not reliably reflect impact of research at society level [20].

Additionally, it has been recognised that ensuring effective dialogue between the research community and citizens is one of the important aspects for catalysing societal impact [21]. It is also believed that qualitative assessments of researchers, experts and citizens (via questionnaires, surveys, interviews, etc.), in combination with quantitative indicators, represent valuable tools to measure societal impact of research [18].

Important monitoring initiatives have been undertaken by the European Commission (EC) in preparation for the post-2020 Programme (Horizon Europe 2021–2027). An indicator framework has been built around a set of Key Impact Pathways indicators, which are meant to monitor the Programme performance towards scientific, societal and economic/innovation impacts within a short, medium and long timeframe [22]. This approach is expected to improve the monitoring and evaluation of the FPs based on the latest technological developments.

As an additional contribution to these activities, the Joint Research Centre (JRC) of the EC, together with the EC Directorate General for Research and Innovation (DG RTD) collaborated on the definition of indicators to retrospectively measure societal impact of EU-funded research in the fields of AD and dementia, BC and PC. Another relevant aspect taken into consideration in this monitoring activity is the understanding of how the selection of the methodological approach may have influenced the translation of research results into societal impact/innovation.

These research fields were selected also considering the large number of animals that are used in related research activities. Indeed, as indicated in the 2019 report of the number of animals used for scientific purposes [23], basic and translational/applied research on the nervous system (including AD and other types of dementia) and oncology (including BC and PC) are among the fields that accounted for the highest numbers of animal uses (22% and 14%, respectively in basic research).

A first analysis of the 202 replies received in the survey was published in a Factual Summary Report [24], and the more recently published Synopsis report [25] provided a more detailed analysis of survey results, complemented with insights obtained from 29 structured interviews to survey participants. This in-depth analysis investigated in particular (i) what type of impact EU-funded research contributed to in relation to the EU FP, the area and field of research, and the selection of the models; (ii) what challenges were encountered, including possible issues with follow-up funding; (iii) what ingredients have contributed to research success and the generation of impact; and (iv) the importance of public engagement.

Here, we discuss the main findings of this retrospective analysis and propose a list of priority actions that could help improve translatability and societal impact of biomedical research.

Ultimately, this retrospective analysis and case studies could provide some important insights on the factors possibly contributing to research success and the obstacles hampering or preventing translational outputs of funded research. Eventually, this may serve as an evidence base to inform research funding bodies in their decision making.

## Methodological approach

### Survey: design and analysis

The survey was conducted using the EU’s survey platform EUSurvey [26] from 14/02/2020 to 31/03/2020. About 3460 emails were initially sent to EU FPs’ participants in the areas of AD, BC and PC; contact details were retrieved via the EC portals CORDA and CORDIS. Additional invitations were sent via email to relevant scientific societies, to enlarge the number of participants, and the survey was shared in social media platforms (e.g., LinkedIn, Twitter), and through the website EU Science Hub. A total of 202 participants (as of 31 March 2020) replied to this survey. Survey analysis was conducted using the free software R analytics (https://www.r-project.org/), and customised scripts were developed to perform a systematic semi-quantitative and cross-dimensional analysis of survey replies. Several survey questions were multiple-choice types and respondents could select more than one reply, which was reflected in the total count of replies.

### Structured interviews: design and analysis

As a follow-up to the survey, 29 in-depth and structured interviews were conducted with a group of survey participants who expressed the interest to be further contacted. Questions of structured interviews were developed to further explore some topics of the survey and get additional insights and explanations from individual participants (Additional file 1), also looking for connections about the different themes. The main themes explored in the interviews were major research outcomes and their social impact, possible translatability issues, funding as a possible challenge and follow-on activities and dissemination to the public.

Interested participants received a formal email invitation and an information sheet describing the background of the study and the investigator team. They were asked to sign a consent form and were provided with contact details for further information.

The majority of the interviewees were in the age group 46–65 (55%) and worked for H2020 projects (60%). No participants working in FP5 projects expressed interest to be interviews and were thus not followed up any further. Moreover, participants selected for interviews were chosen among participants of different projects to obtain a representative panel across research fields and EU FPs. The structured interviews were conducted through video conference system (Zoom or Webex) by three interviewers between May and December 2020. The length of the interviews ranged between 30 and 45 min. At the beginning of each interview, a brief introduction on the scope of the interview and the study was provided. Interviews were audio-recorded and transcribed verbatim.

The coding analysis of interview transcripts was performed by three investigators using a common set of predefined codes (as described in Annex 2 of [25]) by using the software NVivo (version 1.0, https://www.qsrinternational.com/nvivo-qualitative-data-analysis-software/home). Additional file 2 presents an overview of the coding tree with the list of “parent” (main themes) and “child” codes (subthemes) used to analyse the interview transcripts. The coding structure was developed to cover the following main themes: type of EU project Call, disease, dissemination, dissemination means, funding, impact, methods, patents, risk prediction/prevention, diagnosis/patient stratification and translatability. The qualitative analysis of transcripts followed these categorisations and an iterative and crosscheck strategy was adopted to ensure a harmonised and unbiased approach among the three investigators. To confirm the accuracy and validity of the analysis, the investigators identified a series of excerpts from the transcripts to support main recurring themes. Further details about this qualitative analysis are provided in [25].

### Results: main findings and possible strategies to improve translatability of biomedical research

The main findings of this survey and in-depth interviews are presented in the following sections. The outcomes of the survey and interviews provide a valuable starting point to engage with a variety of important stakeholders to reflect on the *status quo* and explore what strategies could be devised to foster human relevance and translatability of biomedical research outcomes and to maximise impact on public health. Such results allow to gain insight into and understanding related to: (i) how EU-funded projects have contributed to innovation and major scientific breakthroughs; (ii) how scientific results have translated into effective societal impact; and (iii) what scientific methods and research approaches underpinned the advances made.

### Most respondents feel their research will have an impact and time is an important factor in the generation of societal impact

Measuring the impact of research is a complex topic especially when looking at the societal aspects of scientific research [27], which entails assessing how publicly sponsored research had a return on culture, society, environment and economy [28, 29]. Evaluation of inputs, activities and intermediate outputs is potentially qualitative, although may account for some quantitative estimates (e.g., the amount of funding). The analysis of the impacts is fundamental to establish how the outcomes have substituted for (or ameliorated) activities and outcomes previously undertaken by others [30].

According to the surveys replies, only 5% of respondents considered that the research activities they received funding for, did not have an impact beyond their project. In the survey, societal impact was defined as ‘*any output that already had, or could have, a tangible impact on public health, specific patient groups, or society at large*’. Despite this definition, we realised during the in-depth interviews that survey respondents often interpreted the term ‘impact’ in a broad sense, considering both scientific and societal impact.

**Fig. 1:**
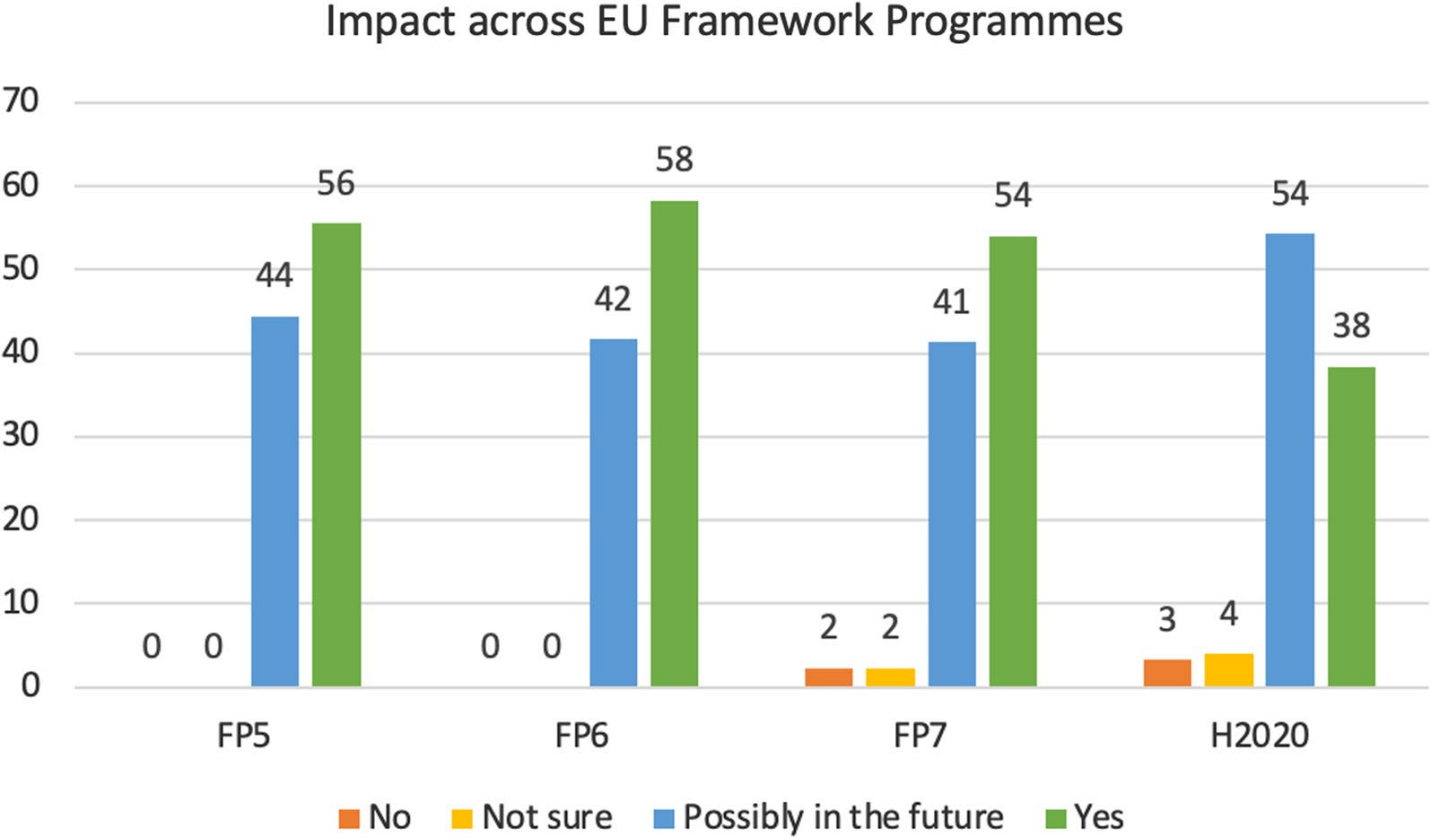
Level of impact in relation to the EU framework program. ‘Yes’ = research had a concrete impact; ‘Possibly in the future’ = an impact may be seen in the future; ‘Not sure’ = impact is not sure; ‘No’ = research generated no impact. Values are shown as percentages of respondents within each FP group

Overall, 40% of respondents indicated that their research had an impact beyond their project, while 53% claimed that an impact may be seen in the future. In particular, 56% of respondents under FP5, 58% under FP6, and 54% under FP7 declared that their research achieved an impact (Fig. 1, green bars). Regarding H2020 programme, which covered the 7 years from 2014 to 2020 and therefore was still running during this analysis, 38% of respondents claimed their research already had a concrete impact, while 54% claimed a possible impact in the future (Fig. 1). The percentage of replies indicating ‘no impact’ was very low, ranging between 0 and 3% across all the FPs (Fig. 1).

Societal impact is difficult to measure as it is not static and can evolve over time. It is therefore easy to understand that most of the respondents chose “Possibly in the future” rather than “not sure” as an option. While the “no impact” response would possibly be indicative either of project failure or no expected outcomes, the “Yes” option shows the manifestation of concrete impact already.

For older FPs (FP5 1998–2002, FP6 2002–2006, FP7 2007–2013), at the time of the interviews—which were undertaken in 2020—adequate time had elapsed since the completion of the project, and therefore it was possible to assess any effective impact beyond the project. For H2020, the temporal dimension impacted the replies and should be taken into consideration in the data analysis (materialised impacts may ultimately be higher in the coming years than reported in the survey). Notably, time lag between scientific research and impact on society can vary considerably and depends on many factors like the type of project or the development of a certain technology [31].

This was illustrated in the interviews by different quotes such as: *“Often people see impact in quite a narrow way and say, ‘Yes okay in three years you will have some sort of drug for this’. Sorry”*, *“When this will have an impact to the patients. To be honest, I think that we’re still very far from it”* or *“To really find its way into, let’s say, clinical practice or really affect the general public, that’s going to be a bit down the line”*.

### Obtaining follow-up funding when needing to continue research is often an issue

**Fig. 2:**
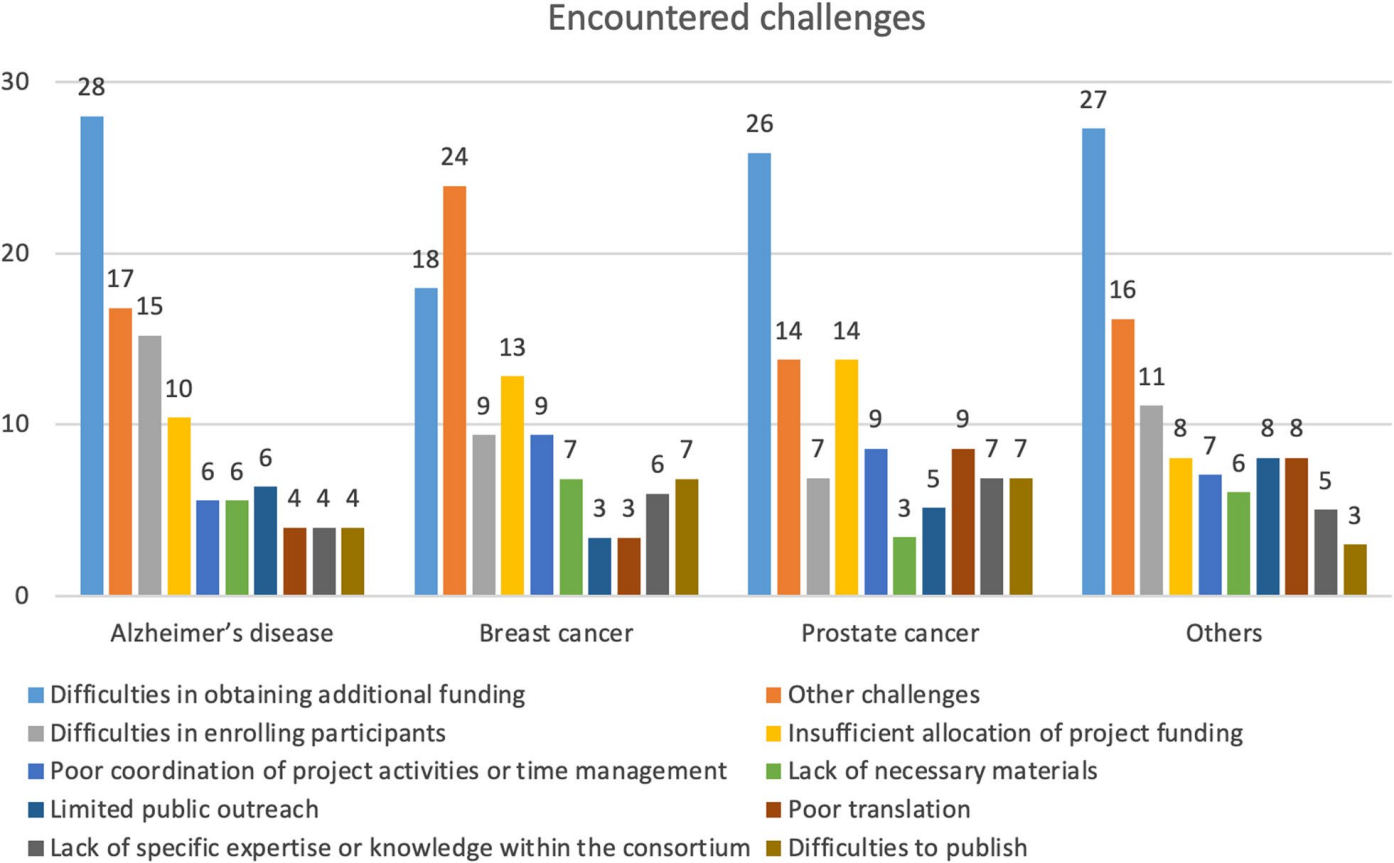
Encountered challenges across different fields of research. Values are shown as percentages within each field of research

We explored the relationships between the encountered challenges and the respective field of research (AD, BC, PC or other diseases) to understand how these challenges may have influenced the research activities and the generation of impact. Twenty-eight percent of people who worked on AD, 26% of those who worked in PC and 18% of those who worked on BC had difficulties in obtaining additional funding at the end of the funding cycle (Fig. 2). Moreover, the difficulty to enrol participants, the insufficient allocation of project funding, as well as poor coordination of project activities or time management, represented three other popular challenges faced by the respondents.

**Fig. 3:**
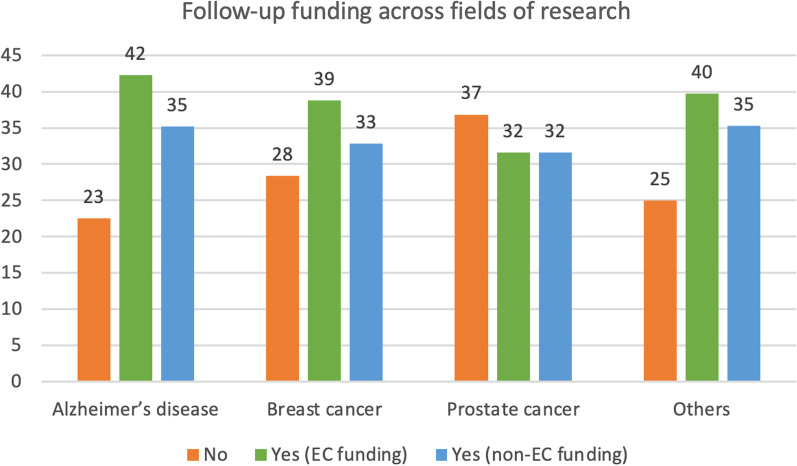
Follow-up funding across fields of research. Values are shown as percentages within each field of research

Concerning follow-up funding, 42% of AD researchers, 39% of BC researchers and 32% of PC researchers succeeded acquiring new EU funding at the end of the funding cycle (Fig. 3, green bars). Notably, many H2020 projects were still ongoing at the time of the survey, and therefore 41% of them selected ‘not applicable’ in reply to the question on follow-up funding.

The possibility to obtain follow-up funding for the most promising projects was commented by some interviewees as a way to improve translatability of research results. The definition of some selection criteria could help decide what projects may be considered as most promising to generate impact.

This was illustrated in the interviews by different quotes such as: *“Often there are no continuation calls, no calls that say we have funded things on these topics four or five years ago, these projects are likely to end right now. There are no opportunities to continue that research because the focus is somewhere else”*, *“For instance, on breast cancer or on comorbidities, there has been a bit of a lack of a follow-up”* or *“That has been an issue in terms of trying to make sure that research can continue smoothly once the project ends”*.

### The major ingredients for a successful outcome of a research project: research strategy design, collaboration with partners and multidisciplinarity

**Fig. 4:**
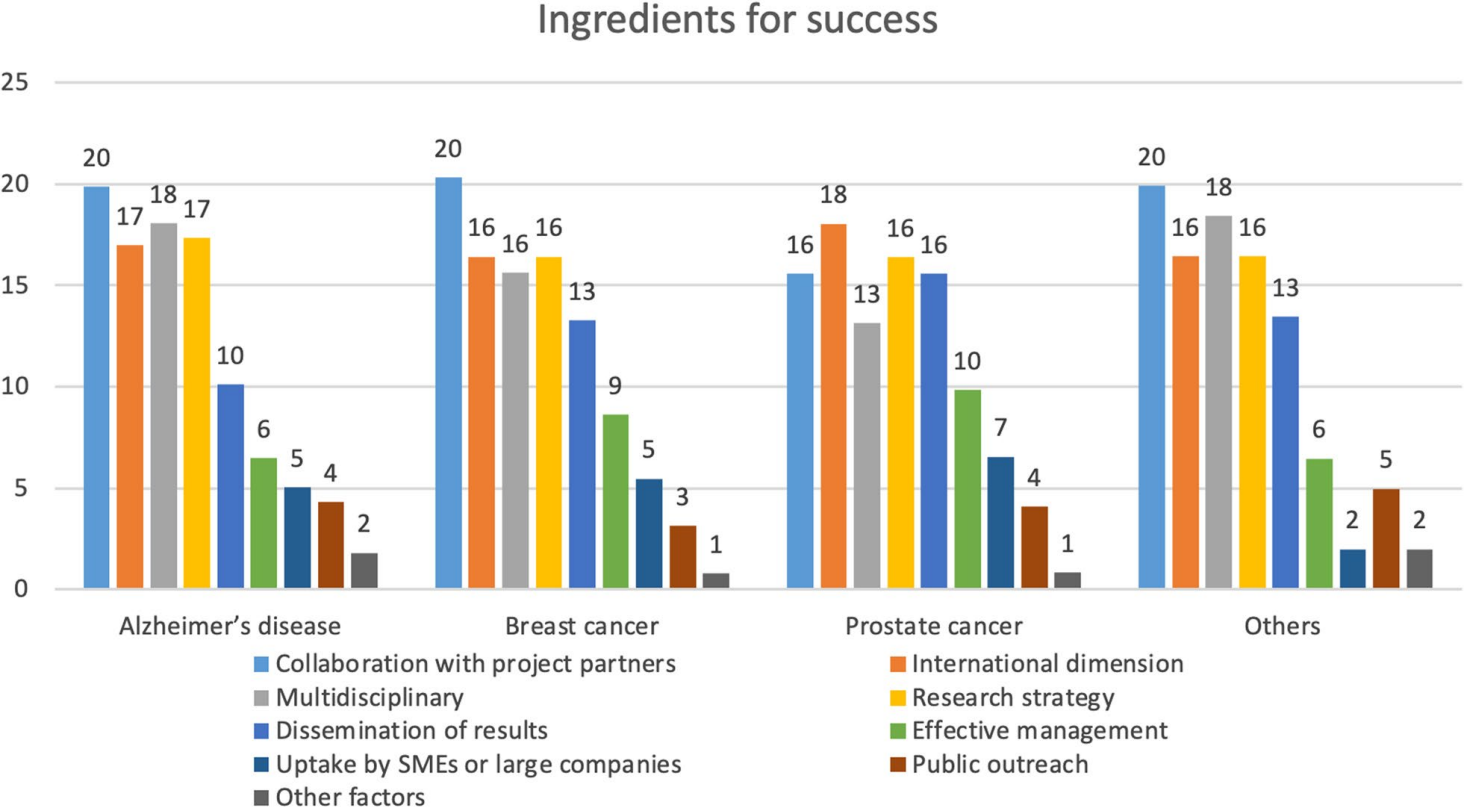
Ingredients for success. Values are shown as percentages within each field of research

Survey respondents recognised collaboration with project partners as a major driver for success of projects, and this was recorded by around 16–20% of participants (Fig. 4).

The international dimension and the multidisciplinary nature of the project, as well as the research strategy were recognised as additional success ingredients by about 17% of respondents (Fig. 4). Notably, 3–5% of respondents considered public outreach as a success factor.

During the interviews, many participants pointed out that nowadays research questions are becoming more complex and require a multidisciplinary research strategy to better tackle them, with broad skillsets in the project team (see participants’ quotes at pages 31–32 in [25]).

As reflected in the survey and the interviews, the main ingredients to determine the success of projects are often closely related and are considered equally important by scientists working in different fields. Collaborative research has become a commonplace and researchers are increasingly required to work across disciplines, institutions and borders [32, 33].

Notably, cancer research is today highly multidisciplinary, with the involvement of several experts spanning from clinicians and molecular biologists to computational biologists, statisticians, and engineers [34]. Similarly, the multifaceted nature of AD and dementia also demands for multidisciplinary approaches [35].

This was illustrated in the interviews by different quotes such as: *“If you want a disease research programme you need multiple models on board, and that includes by the way a few rotations, maybe even modellers, structural biologists who understand the structure of the problems and may be able to predict pharmacological interference points”*, *“I think we are in this transition phase where research becomes more complex and you really need teams with a broad skillset, and you are not going to be able to do very interesting research only with biologists or only with physicists. You really need to have a team that can do many different things”* or *“I collaborate with so many people because I think that one specific individual approach is never enough”*.

### Epidemiology based research has significant potential to generate relevant results

**Fig. 5:**
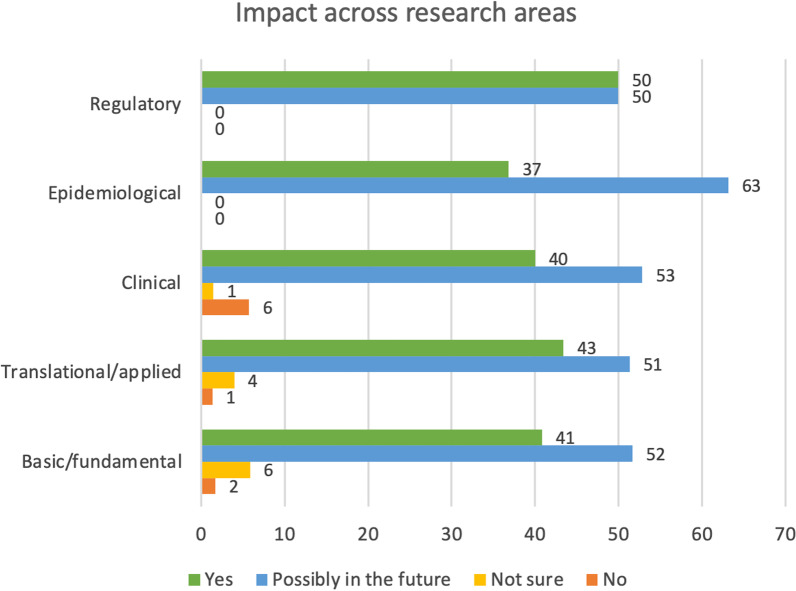
Impact in relation to the type of research. Values are expressed as percentages of replies within each research area

Research impact has been considered not only in a temporal manner, whether it was already gained or possibly achievable in the future, but also in relation to the type of research carried out by the participants. The percentage of people who declared that their research could contribute to generating future impact reached 63% in the epidemiological area and it was a bit more than 50% in basic/fundamental, translational/applied, clinical and regulatory research (Fig. 5, blue bars). The reply ‘no impact’ was generally very low, ranging between 0 and 6% of replies (Fig. 5, orange bars). The number of survey respondents working in epidemiology represented 9% of total participants (19 in 202 respondents), and it was equally distributed across AD, BC and PC research.

This was illustrated in the interviews by different quotes such as: *“There’s going to be less of an issue with translating results you find because those results have been found in actual patients”*, *“There (are) enough biobanks and tissue out there where you can do your actual research instead of circumvent a lot of the translational issues that arise if you try to do it in the animal models”* or *“I think that the quality of research and social impact will increase if some projects could be selected for future translations, and I think that this will also increase the quality of end reports or final reports”*.

### Research aimed at developing novel diagnostic or prognostic tools often leads to more immediate impact

**Fig. 6:**
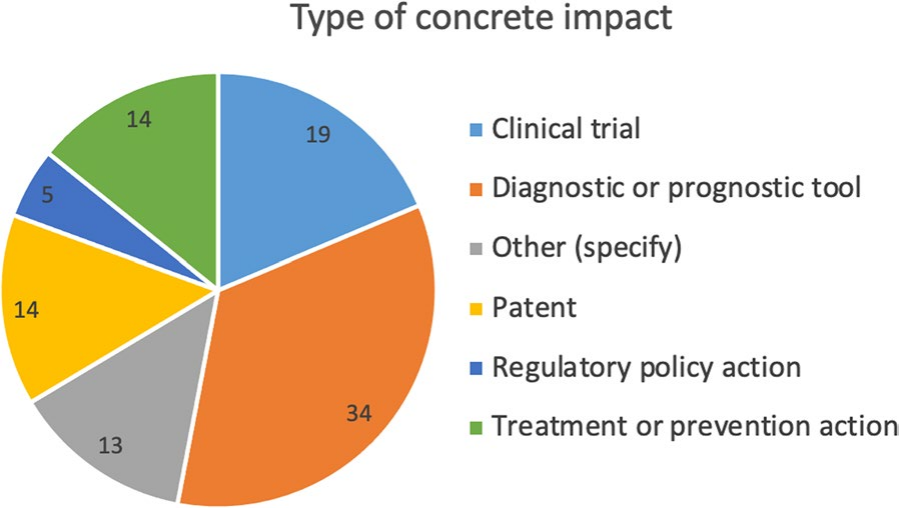
The type of impact beyond the project. Values are shown as percentages within the group of respondents who claimed a concrete impact

Our survey inquired the type of impact that research projects had. Among respondents who claimed that their research had a concrete impact, the development of diagnostic or prognostic tools (34%), the creation of new clinical trials (19%), new treatments or prevention actions (14%), or filing of new patents (14%) were the most selected types of impact (Fig. 6). These types of answers were similarly selected across groups of people who worked in different disease fields and areas of research.

This was illustrated in the interviews by different quotes such as: *“So I think that this is something (that) has big impact, because we can save money for operations, we can improve quality of life for patients and we can avoid unnecessary treatment”*.

Accurate, rapid and targeted diagnosis is critical for identifying the presence of disease at an early stage and for determining an appropriate course of treatment (i.e. prognosis) [36]. Disease prognosis can support in planning care and making decisions about medical interventions, as well as it may help patients to feel more self-conscious about their conditions [37].

### The imp﻿act of advanced in vitro and computational models is increasing with time

In recent years, there has been a shift towards innovative and state-of-the-art models that are not based on the use of animals. Several of these models are mainly based on in vitro, ex vivo and in silico techniques, as highlighted in recent JRC studies that reviewed currently available and emerging non-animal models in several fields of biomedical research, including BC [38] and neurodegenerative diseases [39].

**Fig. 7:**
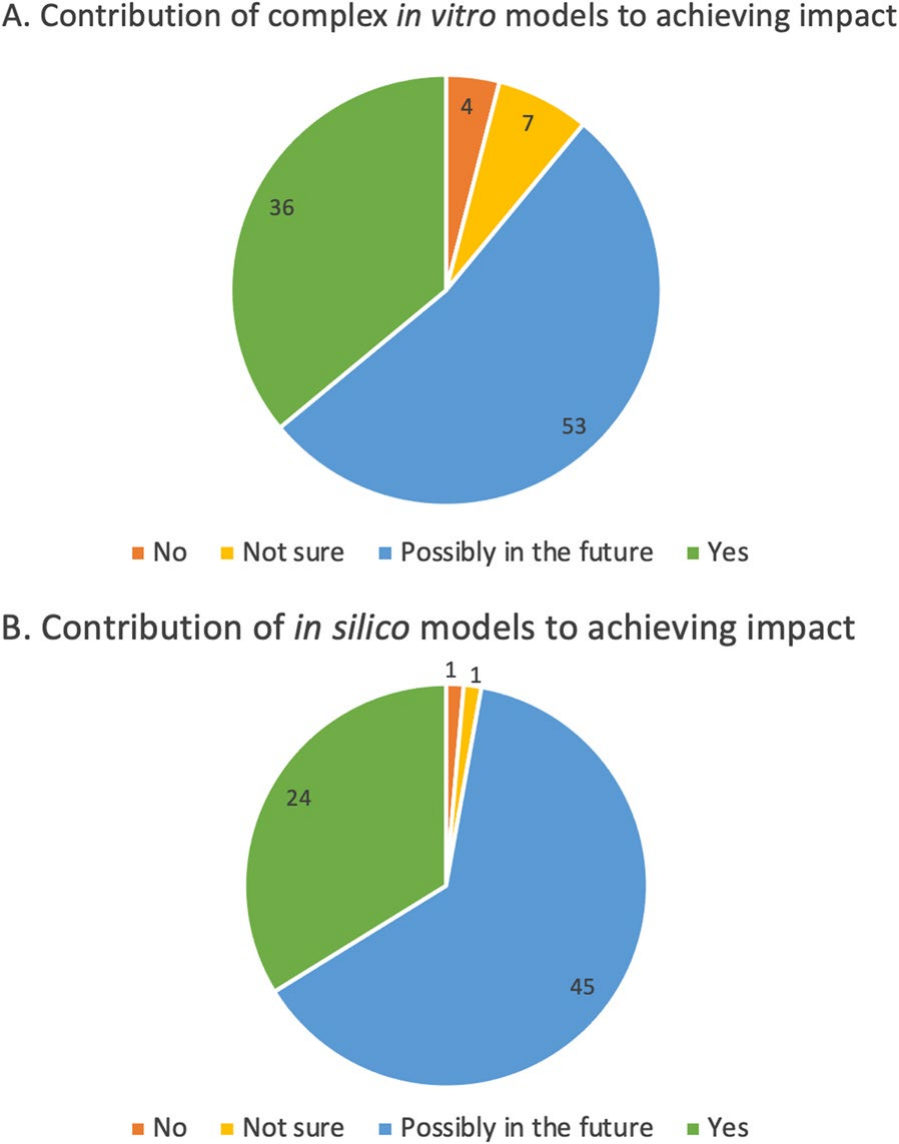
**A** Contribution of complex in vitro models (e.g., 3D models, spheroids, organoids, microfluidic, organ/tissue on chips, etc.) to gaining impact. **B** Contribution of in silico models (in silico/computational models) to gaining impact. Values are expressed as percentages of respondents who considered these models as essential to the success of their project

The survey investigated how complex in vitro models and in silico/computational approaches contributed to the generation of impact. Among users of complex in vitro models, 36% declared that these models were essential to achieve impact beyond the project and 53% considered them as essential to achieve future impact (Fig. 7A).

Of the 60 respondents who used complex in vitro models and considered them as essential to the success of their project, 30% worked on BC, 27% on AD, and 18% on PC (see Annex 3-Table B in [25].

Additionally, 24% of users of in silico/computational approaches declared that the use of these technologies was essential to gain impact, while 45% considered that these approaches will contribute to generate impact in the future (Fig. 7B).

This was illustrated in the interviews by different quotes such as: *“There is development of different kinds of advanced *in vitro* methods like these lung-on-a-chip or brain-on-a-chip type of approaches. Of course, we are also hoping that these kinds of *in vitro* methods could replace some of the animal experiments in the future”* or *“One (important) thing is making a better use of the data because there’s a huge amount of data that it’s being wasted or overlooked so make a better use of data (to) reduce as much as possible the generation of new data when it’s not necessary, and that means using less animals”*.

### The use of animal models is still considered unavoidable by many, despite the recognised translational failures

**Fig. 8:**
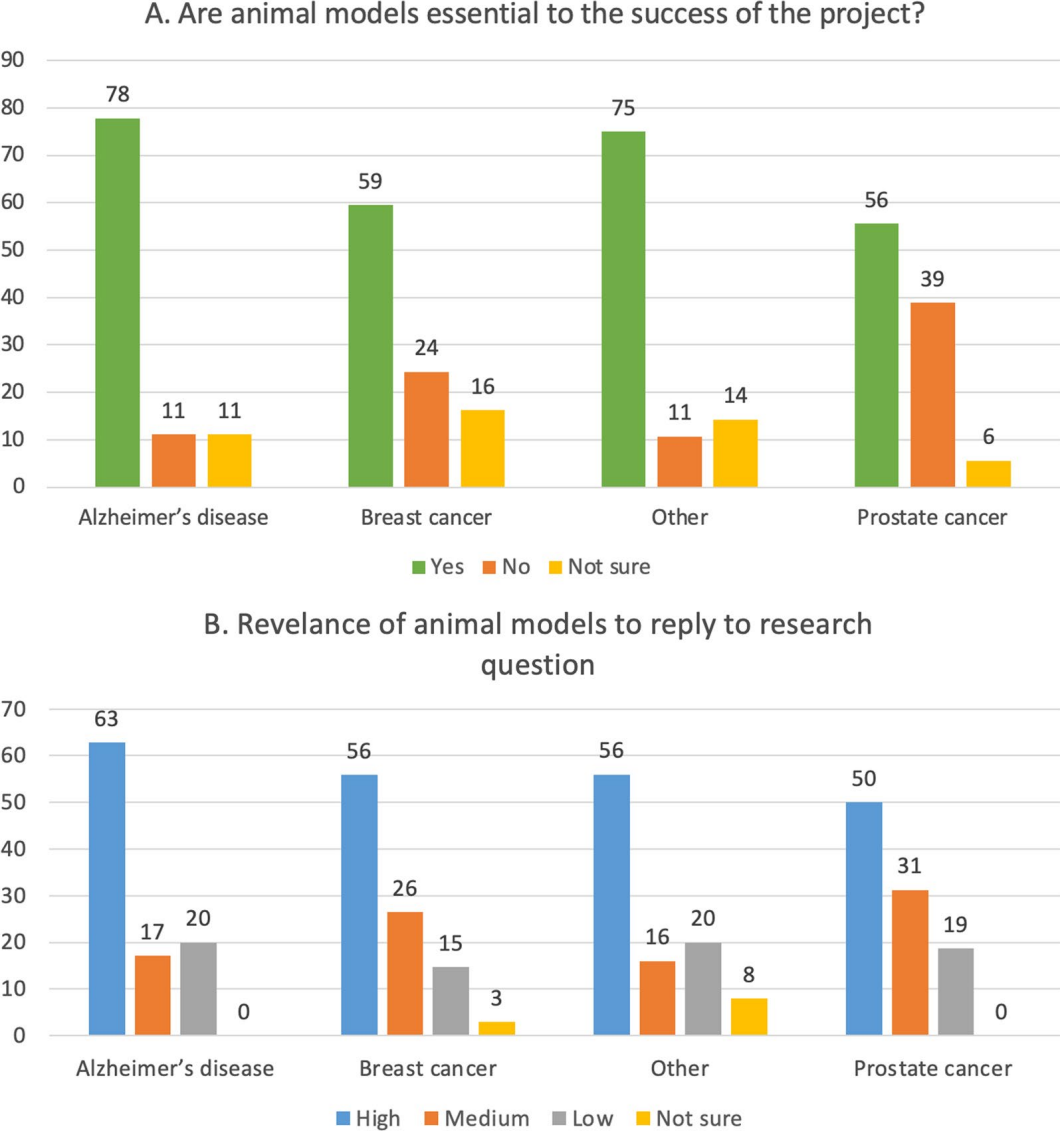
**A** Animal models contribution to research success. **B** Relevance attributed to animal models by respondents who worked on different disease fields. Values are presented as percentages of respondents within each field of research

Between 56–78% of respondents (depending on the research filed) who used animal models considered them as essential to the success of their project (Fig. 8A, green bars). Overall, there was a tendency within the group of scientists engaged in AD research (in comparison to the BC and PC) to consider more frequently the use of in vivo methodologies as essential or highly relevant to their work.

While between 50 and 63% of respondents considered animal models as highly suitable to reply to research question, an average 18% considered animal models as poorly relevant (Fig. 8B).

The vast majority of those researchers who considered in vivo models as highly relevant (27 respondents in total), claimed that their research will demonstrate an impact in the future, while approximately 1 in 3 considered that a concrete impact was already achieved, contributing e.g., to the creation of new diagnostic or prognostic tools (7 in 27 respondents), or new treatment or prevention action (3 in 27 respondents).

Two out of three respondents who used animal-derived material (e.g., cells, tissues or organs derived from animals) regarded them as essential for the success of their scientific projects. On the contrary, 25% of them described them as not essential to the success of the project, and 18% considered these experimental models as poorly relevant to reply to research questions. Moreover, the majority of the researchers who described the use of animal-derived material as essential, claimed that their research either already had a concrete impact (37%) or that it will have an impact in the future (56%) (see Annex 3-Tables A and B in [25]).

According to the responses of the interviewed researchers, the use of animal models is still largely regarded as inevitable due to the following reasons:

- the systemic and complex nature of human diseases, which cannot be delineated (yet) solely by non-animal methods;
- the relatively low cost of maintenance and ease of creating a fit-for-purpose transgenic animal (e.g., mouse);
- the perception that animal models are indispensable when aiming for a high Impact Factor (IF) journal publication or to validate in vitro findings [40].
- the higher chances to acquire funding when animal models are part of the research plan.

This was illustrated in the interviews by different quotes such as: *“I think we’re not there yet for a system like that to replace animal models just because of the systemic nature of many diseases. Ideally, that’s where it progresses. I think that’s at least a decade away”*, *“I think that the rodents are … there is unfortunately weak translatability for human disease. As a preclinical model, we know that it’s not fantastically good, but I think that the cost issue and the fact that you can basically genetically modify animals relatively easily, this is an extremely important tool”*, *“One of the biggest pressures for using different models is what people expect to see in publications. I was told [….] unless you have an animal experiment in your paper, you can only look to aim so high for your impact so I think it’s a little bit the publication culture but also it’s still the gold standard when you’re doing a lot of fundamental discovery work”* or *“I am applying for some grants that can be funded for getting out the animal model work”*.

The general notion is that researchers realise the innate limitations of animal models, since the latter usually cannot recapitulate the complexity of human pathophysiology and—very often—lead to failures when attempting to translate from animals to humans [41, 42]. More specifically, many researchers who took part in our interviews noted that human disease cannot be fully replicated by animal models. Despite these concerns, by looking at overall survey replies, 78% of AD researchers describe animal models as essential for the success of the project, and 63% consider them as highly relevant to respond to scientific questions (Fig. 8A, B).

### Human cohorts and population studies and the use of human specimens are highly relevant to reply to research questions

**Fig. 9:**
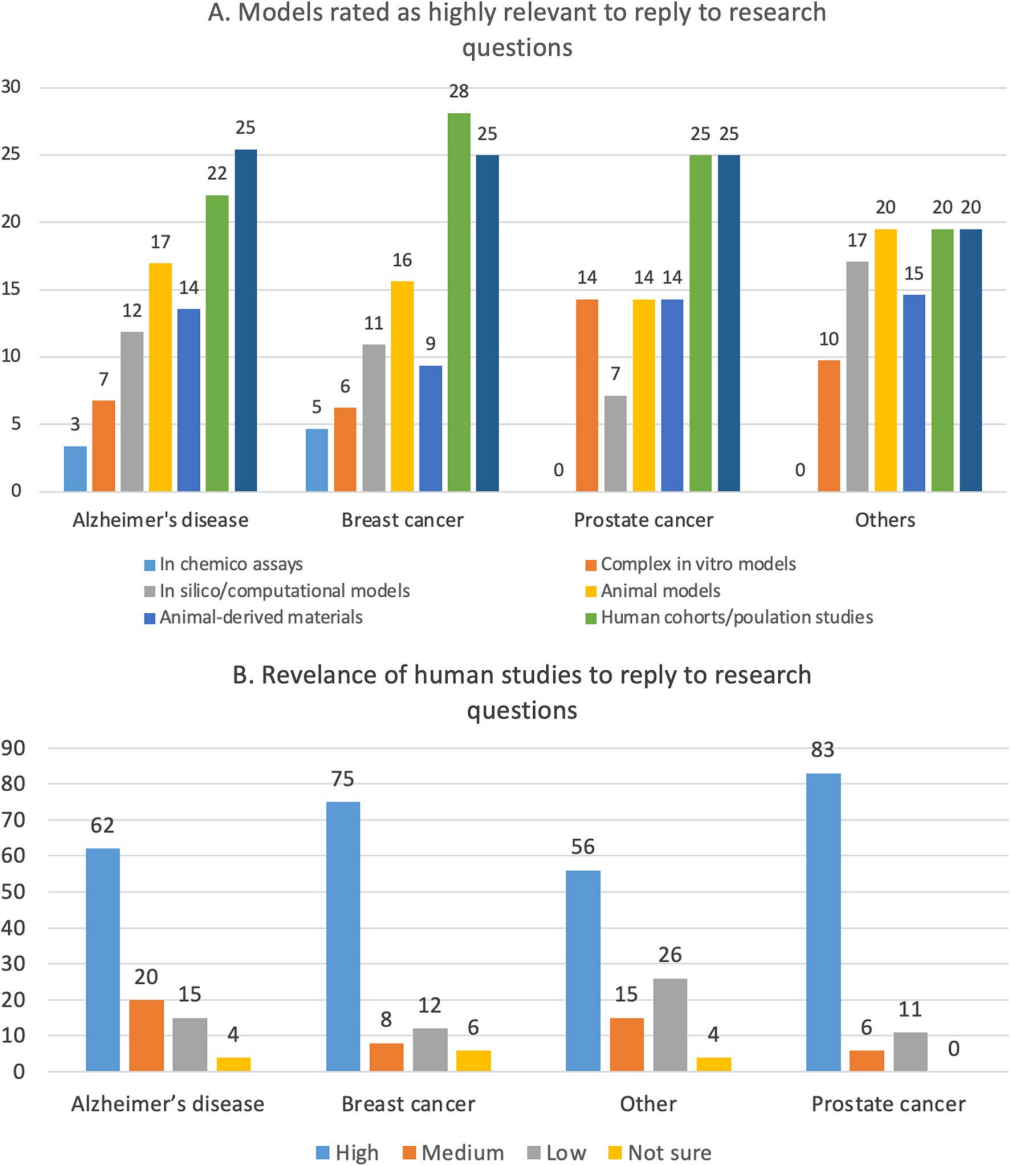
**A **Proportions of respondents within each field of research who considered the indicated experimental models as highly relevant to reply to research questions. **B** Relevance attributed to human cohorts or population studies by respondents who worked on different disease fields. Values are presented as percentages of respondents within each field of research

Between 20 and 28% of respondents within each field of research (AD, BC, PC or other diseases) considered human cohorts, population studies and the use of human specimens (i.e., human-derived cells, tissues, or other biological samples) as highly relevant to reply to their research question (Fig. 9A, green and dark blue bars).

When considering the group of scientists who conducted human cohorts or population studies, an average 68% considered them as highly relevant to their research questions (Fig. 9B, blue bars), whilst an average 16% referred to them as poorly relevant (Fig. 9B, grey bars). Similarly, an overall 70% of users of human-derived materials rated them as highly relevant to reply to research questions [25].

This was illustrated in the interviews by different quotes such as: *“The most important impact here is that what we will do is to test in humans what has been discovered in mice”*, *“Those are patient-derived studies with large cohorts. I’d always opt for those”* or *“I think one of the important reasons for (collaboration) and for success … is always (having) a kind of access to clinical samples”*.

The use of human-based methodologies in biomedical research has been instrumental in the advancements of the recent years. Progress using human samples has shed light onto complex biochemical, cellular and molecular mechanisms governing human (patho)physiology and mediating a wide range of pathological states, which has led to the concept of personalised medicine [43].

It has to be noted that the wider use of human specimens (cells or tissues)—instead of animals—does not come without obstacles. Several legal, ethical and practical issues arise from the acquisition and use of human specimens, which could be discouraging researchers from using them at a broader scale [44].

### It is difficult to enrol participants in clinical studies, especially in the field of Alzheimer’s disease

**Fig. 10:**
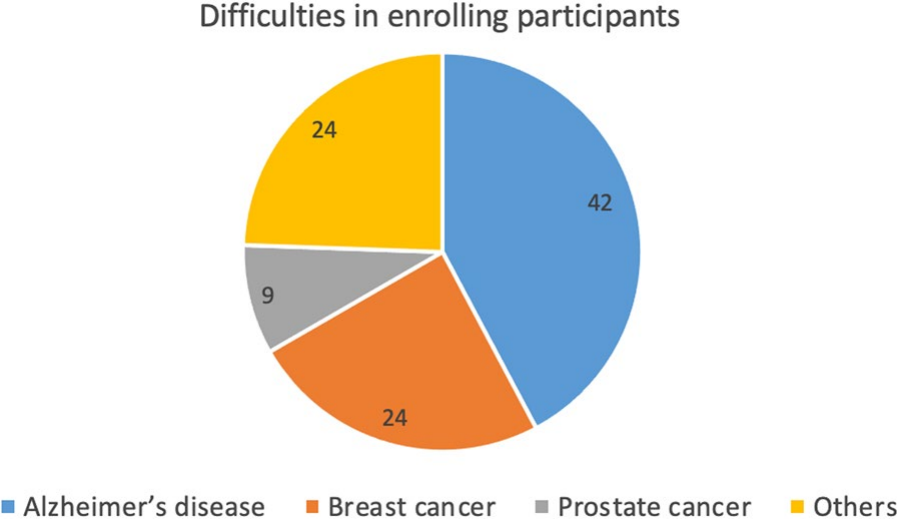
Difficulties to enrol study participants across different fields of research. Values are shown as percentages of each research field within the group of respondents who had difficulties to enrol study participants

Noteworthy, the difficulty to enrol participants was considered as a common challenge by survey participants, particularly by researchers in the field of AD (i.e., 42%, vs 24% of those who worked on BC (or other diseases), and 9% of those who worked on PC) (Fig. 10).

### Disseminating science to the public is important but needs to be conducted properly

The need to involve the society in the scientific debate and the dissemination of scientific results is being increasingly recognised as a mechanism to achieve greater societal impact of funded research, strengthening citizens’ contribution to knowledge in health research, and helping identifying problems and priorities [45].

**Fig. 11:**
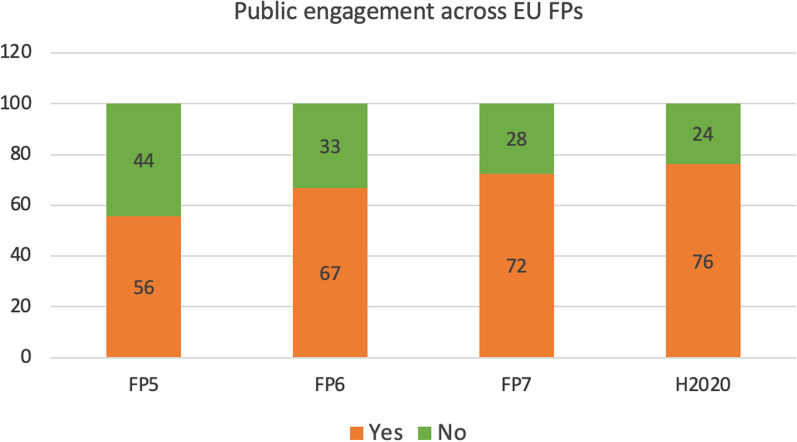
Public engagement initiatives. Values are shown as percentages of respondents within each Framework Programme

Survey results highlighted that disseminating science and results to the lay public was generally considered highly important. In particular, 44% of those who declared their research had an impact beyond their project engaged the general public in several ways (e.g., seminars, online videos, meetings, posters) [25]. Comparing different FPs, these dissemination activities increased over time, as calls for proposals of more recent FPs (FP7 and H2020) explicitly indicated the need to inform the public about project activities and results (Fig. 11, orange bars).

Regardless of the FP, when comparing different research fields, 72–73% of people who worked on any of the disease fields analysed in this study (AD, BC, and PC) referred to lay public dissemination efforts, whilst 27–28% did not make efforts to engage the public [25].

It should also be considered that some participants did not have the time to engage the public, considering that their projects were still ongoing or were at a preliminary phase at the time of the survey. In-depth interviews also helped understanding that among the possible obstacles hampering public engagement, lack of time, especially in the earlier phases of the project (more focused on data generation and peer-reviewed publications) played a relevant role. Other significant concerns were represented by the incapability (or the difficulty) to simplify technological and scientific details, and the risk to raise misleading expectations when communicating scientific results to the public and patients in particular. Some interviewees expressed the opinion that science communicators could (or should) be in charge of dissemination to bridge the gap between science and the lay public [25].

This was illustrated in the interviews by different quotes such as: *“I would say that this kind of funding, which is targeted to find some new solutions to certain diseases and so on, it’s important to communicate also to the public”*, *“If you can’t explain what you’re trying to do to any member of the public, then you’re probably not worth doing what you’re trying to do”*, *“It’s very difficult to effectively disseminate to a lay audience if you’re just doing advances in methodology or advances in detection or characterisation and not treatment”* or *“We need this kind of network of people who can convey the information to the lay people properly, but who at the same time has the scientific foundation to do so”*.

## Discussion

With considerable funding being invested in biomedical research, nowadays it is pivotal to put in place strategies to measure the contribution to innovation and the societal impact of funded biomedical research. In particular, beside advances in understanding the molecular basis of the disease and the progress in diagnosis, prevention and therapy, a high level of translational failure in drug development is observed in these areas, with 97% of cancer drugs [46] and 99% of AD drugs [4, 47] failing to receive regulatory approval. Monitoring research outputs in the context of these diseases is important considering also their high prevalence in Europe and worldwide [1, 2], and the high number of animals used in research activities focused on these disease fields [23].

In this article we discussed the main outcomes of this retrospective analysis, exploring their multiple dimensions. Below we discuss the main findings of this analysis in the context of past and recent activities relevant to the described topics that have been initiated at EU level and introduce some possible suggestions for priority actions that could be considered to foster translatability of biomedical research, particularly in the three disease fields analysed in the context of this activity (AD, BC and PC), and increase societal impact (Table 1).

**Table 1:** Summary of main findings and suggestions for possible priority actions to increase societal impact of biomedical research

Main findings	Suggestions for priority actions
Temporal dimension plays an important role in the generation of long-term societal impact. In particular: 1. Most respondents feel their research will have an impact and time is an important factor in the generation of societal impact; 2. Obtaining follow-up funding to continue research is often an issue	To funding bodies: Accelerate the implementation of calls for proposals to support follow-up research activities aimed at implementing results derived from exploratory research projects, promoting research results using e.g., SME instruments, Horizon Results Platform, EIC, etc
3. The design of the overall research strategy, positive collaboration with project partners, the international dimension and the multidisciplinary nature of the project are considered as the major ingredients for success	To funding bodies and projects’ reviewers: Prioritise projects with a well-thought research strategy and with project partnerships ensuring a broad coverage of multidisciplinary techniques and range of expertise Enable the possibility to look for and hunt for synergies and complementary partnerships to create community networks, overcoming isolation or established reserve due to (funding) competition
4. Research on epidemiology may contribute to generate impact in the long term	To funding bodies: Prioritise calls for proposals focused on epidemiology, prevention, and disease risk prediction. Follow-on funding to continue with these activities should be envisaged
5. Research aimed at designing novel diagnostic or prognostic tools has high chance to generate impact in the short-to-medium term	To funding bodies: Prioritise calls for proposals focused on improvement or implementation of diagnostic or prognostic strategies or tools
6. The use of complex in vitro models and in silico/computational models could contribute to future impact generation	To funding bodies: Design calls for proposals focused on the development, implementation and standardisation of complex in vitro models or in silico/computational models
7. Despite their use is still considered as unavoidable or mandatory, the use of animal models could contribute to translational failures, as commented by some interviewees	To funding bodies: Design calls for proposals incentivising the application of integrated, multidisciplinary human-relevant innovative approaches/ methodologies, downgrading the reliance on the use of animal models
8. Human cohorts and population studies and the use of human specimens are considered as relevant to reply to biomedical research questions	To funding bodies and clinical research institutions: Allocate more resources on multidisciplinary research projects focused on human cohorts and population studies
	To funding bodies, research institutions, biobanks: Subsidise the creation, better use, and transparent sharing of human biobanks (cell and tissue banks) (e.g., as done in the context of rare diseases) Consider multidisciplinary and the important role played by digitalisation for the creation of significant cohorts
9. In the field of AD and other dementias, the difficulty to enrol participants is higher than in the cancer field	To projects’ proponents, research institutions, patients’ associations: Implement strategies to better inform AD patients associations and care givers about the importance to participate in clinical studies
10. Disseminating science to the lay public is important, but communication can be misleading	To projects’ proponents, research institutions: Coordinate and incentivise dissemination activities, e.g. accounting for multidisciplinary, considering involving science communicators in research projects, and training community representatives

### Measuring impact of funded research is a challenging task

One of the main aims of this monitoring activity was to explore societal impact of funded research, which should likely go beyond addressing mortality and morbidity. However, achieving societal impact takes time and it would benefit from being defined earlier and upfront as part of the research design strategies.

There is the need to generate programmes with societal impact that goes beyond the current *status quo* (i.e., aim for inclusiveness-equity-fairness). The most recent Horizon Europe Work Programme 2021–2022 is going towards this direction [48, 49].

Going back to the scientists (about half a million funding recipients) is also a challenging task, as well as understanding exactly who (among different project partners) used the funds and for what part of the project.

Tracing back data and outputs generated within old projects funded under FPs older than FP7 is particularly difficult, as research outputs and data were not closely monitored as it has been undertaken in more recent FPs (FP7 and H2020). Additionally, measuring impact of old projects (e.g., funded under FP5) may be complicated for several reasons, such as—but not limited to—the impracticality to link a certain research output with a specific project, change of career objectives of project coordinators or project partners (e.g., change of job or research topic(s)), etc. On the other hand, assessing impact of more recent projects (e.g., under FP7 or H2020) may also be unfeasible, e.g., lack of sufficient time to move to subsequent clinical or translational phases of research, which could also be linked to lack of follow-up funding, as presented above and commented in the next section. Other online sources could be utilised to get more insights on possible follow-up activities after completion of a project (e.g., data repositories, peer-review publications, patents, creation of spin-offs or SMEs after the end of a project, etc.), and make links between previous funding calls. In line with this approach, the JRC just carried out an activity in collaboration with a DG ESTAT contractor (GOPA, https://www.gopa.de/) aimed at assessing the suitability of a list of indicators to monitor impact of EU-funded biomedical research. Definition of suitable web sources to fulfill indicators data is an integral part of this project, which aimed to perform a more quantitative/objective analysis of funded research outputs, outcomes and impacts, and also understand what factors played a role in the choice of the experimental models (e.g., prior experience, routine, innovation considerations, etc.).

Measuring impact is recognised as challenging considering the time constraints and the gaps in data and information. In lack of gold standard methodologies and indicators to evaluate the impact of research programmes, evaluations that are based on the triangulation of both qualitative and quantitative information from multiple sources, including surveys, in-depth interviews, case studies, expert groups, and econometric analysis can be deemed as valuable tools to retrospectively assess research translation and impact [18].

However, research surveys also present some caveats, as provided information is self-reported or subjective, respondents’ replies may often be different than they might be in other circumstances, and therefore research impact may be overrated [3, 50]. Our survey collected feedback from a relatively small group of participants (202) and followed-up interviews (29) with limited replies from FP5 participants (9) none of whom was interviewed. As this survey was based on voluntary participation, the project coverage was not recorded and several participants of the same project may have replied. This may not support quantitative conclusions about the investigated aspects. However, while keeping in mind these limitations, our monitoring activity (both the survey and the in-depth interviews) have helped us gather a subjective feedback of scientists’ perspective and range of opinions about their experience with EU funding, and how they feel their research may have contributed to societal impact and public health. This has also enabled to reflect on some epistemology-related aspects concerning the decision to opt for the use of a certain methodological approach or model when planning the experimental strategy.

Ideally, impact assessment should move from a qualitative to a more quantitative assessment, making better use of statistics, in order to be able to understand what type of success, what sort of impact, and how such impact can be exploited. It will also be important to understand what environment could contribute to generate research successes or could be conducive to impact. In line with this, the JRC survey investigated these aspects posing specific questions (with multiple-choice replies), in an effort to understand what major hurdles were faced by the researchers during their project and what ingredients mainly contributed to success and the generation of impact.

While time is a crucial factor to achieve societal impact, understanding what happened 10 or 15 years after the end of projects is hardly possible to capture, due to the lack of a systematic monitoring approach in the older FPs, as commented above.

A recent DG RTD activity aims to trace back such impact to project over time; as part of the *Common Knowledge & Data Management Service Unit,* DG RTD is currently mapping analysis of outcomes and potential impact by using a non-intrusive AI-based methodology combined with expert knowledge [51]. First, an extensive list of beneficiaries and participants of the projects (wide list beyond the project coordinators) has been compiled. A wide range of open sources, as well as proprietary resources is being browsed for activities and outputs of project participants over time. These collections of data are being traced back to projects, being included in a knowledge graph system and used as measures of impact. Many indicators—bibliometrics, as well as Altmetrics—are going to be built. The RTD approach is driven by big data and AI, but it might be combined and cross-checked with qualitative inputs (as those collected in the context of the JRC monitoring activity) through targeted surveys and interviews with project participants.

### Follow-on funding to increase the chance of impact

Considering that the temporal dimension plays an important role in the generation of concrete societal impact, the possibility to apply for follow-up funding through dedicated calls for proposals addressed to former funding recipients of most promising research projects could be considered, as commented by the majority of the 29 interviewees [25]. This could enable the possibility to implement results derived from exploratory research projects as, for example, Data 4 Impact [52], EURITO [53] or Intelcomp [54]. In 2017, the call for tender Tracking of Research Results (TRR) was issued and aimed to identify project results emerging after the end of the EU-funded projects [51]. As additional example, the European Research Council (ERC) with the ERC Proof of Concept (PoC) grant scheme supports researchers that have already received an ERC grant (Starting, Consolidator, Advanced or Synergy) for their frontier research and are ready to explore the commercial or social potential of their work [55].

### Impact of epidemiology and human cohorts and population studies

Epidemiology investigates the disease spread, its underlying factors and proposes measures for disease control and prevention [56]. Considering that epidemiology research has high chance to provide societal impact in the long term, studies focused on epidemiology, prevention, and disease risk prediction could be prioritised when evaluating grant proposals for assignment of funding. Epidemiological studies can help identify risk factors and apply a multidisciplinary approach to better understand how socioeconomic and lifestyle factors affect health outcomes [57].

A recent study analysed H2020 funded projects and showed that epidemiological studies represented 5% of the total projects in health and accounted for 14% of allocated funds. These studies covered, for example, disease modelling, strategies for health policy evaluation and data-driven computational models to classify patients, exposures, and outcomes [58].

Moreover, the need to ensure availability and accessibility to high quality data was identified as a key element to empower research projects outputs, as recently discussed [59]*.* There are high quality data from retrospective cohort studies, but many of these studies suffer from being fragmented and the data collected being not systematically curated [60], which can hamper future use and applicability of modelling tools. Therefore, it is important to both map outcomes and impacts, combining agnostic methodologies and expert opinions with the use of high-quality data and statistics.

Human-oriented research and a focus on human data generation through large-scale human cohorts and population studies should be fostered to increase translatability of science. In line with this, most recent calls for proposals have been designed to support research based on the use of new technologies, spanning AI applications e.g., to predict best treatment strategies [61, 62], big data generation, with a focus on human (clinical) data [15].

Most of the survey participants acknowledged human studies and the use of human-derived material as essential for the success of their projects, highly relevant to answer scientific questions and crucial in producing an impact.

Various actions could be implemented, in order to promote the enhanced utilisation of human-based studies and accelerate their adoption by the regulatory authorities. An important action would be to further support multidisciplinary research projects focused on human-based approaches. State-of-the-art human-focused approaches will be key for innovation and the advancement of translational research. H2020 already supported projects employing cells and tissues isolated from human subjects and implementing investigator-driven clinical trials and human cohort studies [63]. Secondly, more weight should be given by funding bodies, research societies, governmental institutions and cell/tissue banks in promoting data sharing, collaboration, trust and multidisciplinary, as well as tackling issues with (im)proper annotation of human samples, with the inclusion of minorities, in order to accelerate the wider use of human specimens in top notch research. Thirdly, community outreach will likely have profound effects in the recruitment of more patients in research.

Additionally, our analysis revealed that research focused on improvement (or implementation) of diagnostic and prognostic strategies or devices seems to have higher chance to generate societal impact in the short-to-medium term. Along this line, Horizon 2020 funding programme has supported research in the diagnostic area with a call on ‘*Clinical research for the validation of biomarkers and/or diagnostic medical devices*’, focused on in vitro diagnostics, clinical validation of new biomarkers and device optimisation with focus predominantly on cancer [64, 65]. The analysis of funded projects allowed to identify emerging tendencies as a broader conception of diagnostics tools, along with prognostics and prevention strategies, in an effort to move towards personalised medicine [65]. Development of diagnostic and prognostic tools [66] will play a key role in the projects and activities under Europe’s Beating Cancer Plan [67].

### Animal models and non-animal approaches

In basic/fundamental and translational research, the selection of the methodological approach(es) and scientific model(s) plays a critical role, as it can influence the way in which research problems are both formulated and addressed [3]. Our retrospective analysis explored this aspect and suggests that projects focused on the application of human approaches, including the development, implementation and standardisation of complex in vitro models, such as 3D models, organoids and organ/tissue on chips microfluidic devices, or computational models, have high chance to generate future impact. On the other hand, the use of and reliance on animal models, although deemed as important in biomedical research in the three investigated fields (AD, BC and PC), may contribute to failures of translational research, as suggested by some interviewees [25].

Human-based models, such as human stem cell-derived organoid cultures, confer important advantages over animal models. They can be established in a fraction of the time required for the development of a transgenic mouse model, they are easier to handle, and they provide faster results, while they supply more abundant material for experimentation and can model human tissue physiology more accurately [68]. However, they do have disadvantages in comparison to in vivo models, as they do not resemble the complexity of a whole organism, and standardisation is largely lacking, which makes their reproducibility and regulatory acceptance challenging [69]. Indeed, scientific and clinical validity is lacking for complex in vitro and in silico innovative methodologies—partially because they have advanced very rapidly—and so they are not adequately standardised, which has repercussions for their use in validation/qualification [70]. Recent initiatives have been undertaken by the JRC to foster standardisation of complex in vitro systems, such as organ-on-chip (OoC) devices [71].

Innovative approaches such as organ-on-a-chip not only have the potential to revolutionise the way research is conducted [70], but also to reduce R&D costs by a range of 10–26%, which could mean saving considerable amount of money in the clinical development of medicines [72]. The European Medicines Agency (EMA) has expressed the will to promote the 3Rs for the development, manufacturing and testing of medicines and has set up a special multidisciplinary group (Innovation Task Force [ITF]), aiming to provide a forum for early discussions on innovative tools and medicines [73]. Special consortia have been set up—like the Transition Programme for Innovation without the use of animals (TPI)—with the involvement of state authorities [74], in order to accelerate the transition to animal-free innovations.

Such a transition in a universal/global scale appears challenging without serious investments. One incentive would be the design of calls for funding that would be focused on the development and/or implementation of complex in vitro models (3D cultures, spheroids, organoids, organ-on-a-chip etc.) [75], as well as in silico/computational models [76, 77] in order to revolutionise drug discovery. This recommendation is in line with the latest (16/09/21) EU Parliament resolution, according to which an *‘increased and targeted’* funding strategy is required, in order to push towards the accelerated development, validation and wider use of alternative non-animal methods [78]. For example, specific calls addressing the use of in silico/computational approaches were already opened under H2020 programme and more recently under Horizon Europe [15]. Recent calls for proposals aim at fostering multidisciplinary projects that use new technologies (e.g., artificial intelligence [AI], big data, etc. [79, 80]) and focus on human/clinical (patients) data [15, 81–86].

### The importance of human data and centralised biobanks

Zooming in on human data is crucial; however, as highlighted in the Synopsis report [25], recruitment of patients might be challenging, as noted in the case of AD. In this regard, curated cohort studies in the EU can enhance readability.

Sharing of human data sets within and beyond the projects has been recently discussed also in the context of UN’s 17 sustainable development goals [87]. Further adoption and improvement of open data sharing policy like those promoted by the European Open Science Cloud (EOSC) (https://eosc-portal.eu/) is considered important for health data generated by research projects.

The platform Health Data Space [88] has recently been created to promote better exchange and access to different types of health data (e.g., electronic health records, data from patient registries, genomics data, etc.), in order to support both healthcare delivery (primary use of data) and health research, as well as health policy making (secondary use of data).

Fostering the creation and sharing of well-structured human cell and tissue biobanks will support the generation of big data and enable precision medicine. In recent years, simple biological sample repositories have evolved into biobanks, characterised by complex units belonging to large infrastructure networks, such as the Pan-European Biobanking and Biomolecular Resources Research Infrastructure (BBMRI) [89].

A 2019 literature review analysis of available biobanks showed that most biobanks have been created to support specific research projects, and therefore evolved in a decentralised manner [90]. Consequently, biobanks generally lack harmonisation of procedures for sample collection, processing, and storage, including remarkable differences in biobank sustainability, provided informed consent models, sample ownership and veto rights. The creation of biobanks is considered as the most promising way to make basic research translating to practical medicine [91, 92].

In recent years, FP7 and H2020 funding programmes supported the development and functioning of biobanks in Europe [93]. International organisations, such as the European BBMRI, play a key role in supporting biobanking initiatives, enabling standardisation of several processes concerning e.g., data acquisition and data analysis, and transparent sharing of biological and clinical data. Modern biobanks should also allow large-scale analysis to enable the identification of specific disease biomarkers using either biological or digital material, complemented with well-annotated clinical and biological data. This ultimately would allow effective biomarker identification and the design of personalised medical strategies [94].

In addition to biobanks, patients’ registries are also playing a pivotal role. In this perspective, patients’ enrolment in clinical, observational and interventional studies is crucial. However, as highlighted by several survey participants, it is often difficult to enrol participants in clinical studies, particularly in the field of AD and dementia research [25].

Enrolment of AD volunteers and their health care providers to participate in clinical trials is often hampered by several obstacles. These include health carers’ lack of time, unavailability of diagnostic clinical tools, presence of patient comorbidities, possible concerns over risks to AD patients of experimental protocols, and difficulties to reach a research centre [95, 96]. Additionally, it has been shown that physicians are often not aware of cognitive impairment in more than 40% of their cognitively impaired patients [97], which could also be associated with the fact that healthcare organisations hold different opinions over the value of clinical screening for cognitive impairment. Moreover, lack of awareness of research opportunities by physicians, and concerns about referring elderly patients represent additional barriers to patient recruitment [98].

Several strategies could be considered to increase enrolment of AD patients in clinical trials [99]. In particular, communication and interaction between partner researchers, clinicians and patients’ associations should be improved e.g., by informing physicians about planned or ongoing local trials and granting access to research experts. This is essential to build trust, increase awareness about opportunities for research participation, and design trials with an inclusive approach [95].

Moreover, existing registries should be better connected and coordinated, such as the European network of cancer registry (https://www.encr.eu/jrc), in order to more efficiently enrol the right participant in the right clinical trial at the right time and at the nearer location [100, 101].

### Communication with the public

Considering the aforementioned aspects, communication with the public and in particular with patients and patients’ associations, health care providers, and biobanks participants is key to ensure dissemination of new research programs, research projects, and eventually the uptake of scientific results, enabling to bridge across different communities.

The potential users and “consumers” of the scientific outputs should be taken into consideration already from the design of project calls. It would be important to both co-design projects (and project calls) and co-create research outputs with the end-users (e.g., patients’ associations, caregivers, or other researchers), and this could be achieved by establishing a collaborative environment and cooperation between researchers and end-users. In this perspective, patient associations’ and caregivers’ opinions could inform the follow-up of funding strategies.

As commented by several interviewees, public engagement is important, and most of them considered such engagement activities as rewarding experiences. However, some of them also highlighted the possible associated concerns, such as the difficulty to simplify technical scientific language, the possible risk to be misunderstood or misinterpreted, or even the risk to raise expectations about the possible availability of novel (although still under investigation) pharmaceutical compounds.

While some interviewees commented that science communicators could help bridge the gap between researchers and the general public, the role played by trained community representatives could also be important [102]. However, public engagement entails time and financial resources to be done properly, something that funding bodies would need to recognise. On the other hand, research institutions should also recognise the importance of mentoring and training their researchers, enabling them to acquire good communication skills, for example by organising communication training courses, or hosting institutional events, such as ‘open days’, to allow the public to meet the scientists, as witnessed by several interviewees. Effective and well-planned public dissemination strategies will also increase societal impact of research projects. Public engagement has increasingly become a prerequisite of several recent calls for proposals under FP7 and even more under H2020 FP (as shown in Fig. 11).

### Additional strategies to promote research projects’ implementation

The dynamic feedback from projects results to policy and back, is very important, as well as the creation of a portfolio of best practices that, for example, could help optimise some calls for proposals. The creation of a knowledge hub or Wiki-like platform could be envisioned, aiming to coordinate and share successful examples of projects, both informing and collecting inputs from (end) users. It should also be considered that most scientists are not aware of the results of other scientists, and these tools/platforms, such as the Horizon Results Platform (HRP) [103], could increase knowledge sharing.

Moreover, marketplaces could speed up the uptake of these tools. For instance, a very recent 2021 Marketplace online event focused on best practices in risk factors of non-communicable diseases [104].

The Best Practice Portal [105] has been designed to ease finding reliable and practical information on implemented practices recognised as the best in the area of health promotion, disease prevention, and the management of non-communicable diseases. It also provides an overview of practices collected and transmitted in actions co-funded under the Health Programmes. Other best practices, more linked to secondary prevention strategies (e.g., at what age undergoing mammography, what type of screening machines should be preferred, general recommendations for BC or PC management, etc.) can be implemented by Member States in a voluntary manner.

DG SANTE is also collaborating with the Organisation for Economic Co-operation and Development (OECD) on the implementation of the Best Practice project; in particular, they are building a methodology to sustainably and efficiently filter and present objectives to Member States, helping them to implement Best Practices and identify implementable results [106].

On the other hand, DG RTD is working on classifying and clustering projects in a machine-readable portfolio. Building these tools can help identify those projects that will be invited to share ideas and promote themselves on a research platform. Pitch events are also organised to present projects to investors with almost a one-to-one discussion.

### Other international monitoring activities

Similar to our retrospective assessment activities based on the use of indicators, the Translational Research Impact Scale (TRIS) was built to provide a systematic approach to measure the short- and long-term impact of research activities. This was done by using 72 indicators organised into three main impact domains and nine subdomains (i.e., research direction and resources, research management and conduct, research methods, research results, research dissemination, translational impacts, policy development, community improvement, and consumer resources and behaviour), which were validated by expert groups [107].

Other surveys have been conducted by other international institutions, in an effort to monitor translatability, innovation and impact of biomedical research. For instance, a best–worst scaling analysis survey addressed to both UK Medical Research Council grant-holders and general public representatives aimed at assessing how beneficiaries and producers of research value different kinds of impact. The survey results showed that improvement of life expectancy, the creation of new jobs and the reduction of health costs were considered as main research impacts [108].

A Spanish survey addressed to researchers working in hospitals and research centres affiliated with the Spanish National Health System (NHS) indicated that involvement in clinical research is generally associated with increased scientific output and has a high impact on scientific productivity (i.e., number of articles in high-impact journals) [109].

Notably, a large-scale survey with more than 9,000 researchers, aimed at exploring societal relevance of research, as part of a project the Springer Nature group is undertaking with the Association of Universities in the Netherlands (VSNU) [110], whose results have been reported in a series of blogs. Interestingly, 48% of respondents declared that they considered societal impact before carrying out their research in collaboration with their co-authors or supervisor, whilst only 14% discussed intended societal impact with their funding body’s contact points [111]. Conference participation and the use of social media were considered as the best ways to increase societal impact [27]. However, while participating in scientific conferences and using social media platforms can clearly contribute to academic impact and help reach out research communities beyond personal networking groups, their effectiveness in enabling general public engagement is highly debatable. The analysis also showed that the top priority for most researchers is to reach (be read and cited by) other researchers within the same field of research, and some respondents did not make a distinction between academic impact and impact beyond academia. Moreover, lack of time and of a comprehensive approach or methodology to measure societal impact were considered as the main barriers hampering the assessment of societal impact [112].

Table 1 summarises the main findings of the survey (left column) and presents a list of possible priority actions (right column) that could help improve translatability of biomedical research. These actions could be considered as a starting point to engage with a variety of important stakeholders (e.g., funding bodies, research institutions, patients’ associations, projects’ reviewers, biobanks) and explore collectively possible strategies to foster human relevance and translatability of biomedical research outcomes and maximise impact on public health.

Noteworthy, the actionable points presented in Table 1 are present also in the Cluster 1: Health under Horizon Europe [113]. Topics like prevention, disease risk prevention, data integration and use of AI for personalised medicine are being very well reflected under the health work programme 2021–2022 [114].

## Conclusions

Measuring societal impact of biomedical research is an extremely complex and challenging task, entailing a combination of both quantitative and qualitative approaches. Our retrospective assessment of EU-funding contribution to scientific breakthroughs and societal impact, along with the activity on data gathering by means of defined indicators, could help shed some light on the major factors contributing to making biomedical research more impactful, to better inform policy and address societal needs. In this context, the design of the research methodology can play an important role in determining translational impact of biomedical research, especially in the three disease fields under investigation (AD, BC and PC), as it can influence the way in which research problems are formulated and tackled. In particular, our analysis showed that research focused on human subjects and the generation and sharing of human relevant, large clinical data sets may have great potential to generate societal impact. In addition, crossdisciplinarity and better communication should be fostered to bridge the gap between scientific research and clinical practice, between scientists and the general public. To this end, proactive collaboration among different EC DGs, Agencies and Member States, complementing different expertise and interests is important to match qualitative and quantitative information stemming from the impact building cycle, accelerate the development and the uptake of human-relevant methods, and incentivise progress particularly in the field of biomedical research, as recently advocated also by the European Parliament [78].

## Supplementary Information

**Additional file 1**: Interview questions.

**Additional file 2**: Description of the coding tree with illustrative quotes from the interviews.

## Data Availability

The datasets generated from the survey “Monitoring Innovation and Societal Impact of EU Funded Research” [26] and analysed during the current study are not publicly available due individual data protection as described in the privacy statement (https://ec.europa.eu/eusurvey/files/b58b1fdc-4d61-4e0b-a52a-a41915dee598/add4e355-8be0-409f-83a2-4f851ef902de). Aggregated data are available in a factual summary report [24] and a synopsis report [25].
