# Supplementary material for: Gauging innovation and health impact from biomedical research: survey results and interviews with recipients of EU-funding in the fields of Alzheimer’s disease, breast cancer and prostate cancer
Source: Health Res Policy Syst. 2023 Jun 29;21:66. doi: 10.1186/s12961-023-00981-z (PMC10308747; doi:10.1186/s12961-023-00981-z)
Supplement: Supplementary file 1 — Additional file 1: Interview questions. [file 12961_2023_981_MOESM1_ESM.docx]

**Additional file 1. Interview questions** (conducted online and recorded using Webex or Zoom).

*Note: questions were related to Survey questionnaire (indicated in blue) and replies provided by participants.*

Date of interview and time:

Name/last name: (recorded but kept confidential/undisclosed)

Age: (recorded but kept confidential/undisclosed)

email: (recorded but kept confidential/undisclosed)

| **Main theme 1: major research outcomes and their social impact** |
| --- |
| Survey Q8: Did these outcomes have impacts beyond your project(s)?  Consider what the participant replied (‘yes / no / possibly in the future’), and possible additional comments.  Survey Q9: What type of impact did (your major scientific outcomes) have?  Check his/her reply(ies).  *Goal: enquire how those outcomes have translated (or will translate) into some practical outputs that have (or will have) social impact; if no impact was claimed, ask the reasons behind this.* |
| **Interview questions** |
| 1. (if they replied ‘yes’): Can you explain how your research outcomes have translated into some practical outputs that had social impact? |
| 1. (if they replied ‘possibly in the future’): Can you explain how your research outcomes will translate into some practical outputs that will have social impact? |
| 1. (if they replied ‘no’): Can you explain why your research outcomes had no social impact? |
| **Main theme 2: possible translatability issues**  (Note: by translatability we mean the transfer of basic in vitro and in vivo research into human applications) |
| Survey Q11: What were the most significant challenges?  Check his/her reply(ies). Note the 'translatability issue' was an option.  *Goal: (regardless if he/she has indicated ‘translatability’ as an issue or not) enquire whether he/she considers lack of (or poor) translatability as a relevant issue in biomedical research*  Survey Q17a. How relevant was the model used to the research question(s)?  Survey Q17b. Was the use of this model essential to the success of the research?  Check his/her reply(ies). Participant may have often selected multiple models (‘not applicable’ means that that model was not used).  *Goal: enquire whether the selection of the models can impact (directly or indirectly) translatability* |
| **Interview questions** |
| 1. (if they indicated ‘translatability’ as an issue): Can you explain what factors may have limited translatability of your research? 2. According to you, what other factors may influence translatability in biomedical research? |
| 1. (if they did not indicate ‘translatability’ as an issue): Do you consider lack of (or poor) translatability as a relevant issue in biomedical research? 2. According to you, what are the major factors that may influence translatability in biomedical research? |
| 1. (to ALL, if this aspect is not already considered in the above questions): Can the selection of the models/methods have an impact (directly or indirectly) on translatability? 2. If yes, in what ways? |
| **Main theme 3: funding as a possible challenge and follow-on activities** |
| Survey Q11: What were the most significant challenges?  Check his/her reply(ies). Note that lack of (or insufficient) funding were possible options.  Survey Q15: Within two years after the end of the project, did you receive additional funding to continue with the research activities?  Check his/her reply(ies), in particular check if he/she said ‘no’ or ‘not applicable’  *Goal: if funding was an issue and she/he didn’t receive additional funding to continue with her/his research, enquire what she/he did next and using what methodological approach.*  (Link this aspect also to Survey Q16. Did you change strategy, model or methodological approach in your subsequent research projects? Check his/her reply(ies)) |
| **Interview questions** |
| 1. (if they indicated ‘funding’ as an issue): Can you tell us what type of research activities or topics did you work on at the end of your project? 2. What type of methodological approach did you use? |
| **Main theme 4: dissemination to the public** |
| Survey Q13: Did you engage the general public (i.e., lay audience) to disseminate these results? Consider what the participant replied (yes / no), and possible additional comments.  *Goal: enquire about* *additional aspects of this dissemination/engagement effort(s), or, if not done already, how he/she plans to disseminate those major outcomes in the future.* |
| **Interview questions** |
| (if they replied ‘yes’): Can you explain how you disseminated your research outcomes to the lay public? |
| 1. (if they replied ‘no’): Will effort to disseminate your research outcomes to the lay public be made in the future? 2. If yes, how will you do it? 3. If no, can you explain why not? |

Note any further comments: ________________________________
