# Supplementary material for: Gauging innovation and health impact from biomedical research: survey results and interviews with recipients of EU-funding in the fields of Alzheimer’s disease, breast cancer and prostate cancer
Source: Health Res Policy Syst. 2023 Jun 29;21:66. doi: 10.1186/s12961-023-00981-z (PMC10308747; doi:10.1186/s12961-023-00981-z)
Supplement: Supplementary file 2 — Additional file 2: Description of the coding tree with illustrative quotes from the interviews. [file 12961_2023_981_MOESM2_ESM.docx]

**Additional file 2. Description of the coding tree with illustrative quotes from the interviews**

The table shows the coding tree with main themes and subthemes used to analyse the interview transcripts. Quotes extracted from the interviews are shown to better illustrate some of themes and subthemes.

| **Main theme** | **Subthemes** |
| --- | --- |
| **Dissemination** | effective & important |
|  | possible concerns |
| **Dissemination means** | Public events-debates |
|  | Social media |
|  | Videos-websites-others |
| **Funding** | follow up |
|  | issues with follow up |
|  | past or current |
| **Impact** | early-new scientific knowledge |
|  | future or potential |
|  | personal and professional |
| **Methods** | animals |
|  | development |
|  | human based-non animal |
|  | multidisciplinary |
| **Patents** | not patented |
|  | potential or future |
|  | submitted |
| **Project calls** | FP5 |
|  | FP6 |
|  | FP7 |
|  | H2020 |
| **Disease** | AD dementia |
|  | BC Breast cancer or other women cancers |
|  | PC Prostate cancer or other men cancers |
| **Background** | *in vivo* |
|  | other - non *in vivo* |
| **Diagnosis- patients stratification** | - |
| **Risk prediction-Prevention** | - |
| **Translatability** | concerns with animal models (scientific & ethical) |
|  | relevance of animals-still needed |
|  | success-not issue |
